# Supplementary material for: N-type fast inactivation of a eukaryotic voltage-gated sodium channel
Source: Nat Commun. 2022 May 17;13:2713. doi: 10.1038/s41467-022-30400-w (PMC9114117; doi:10.1038/s41467-022-30400-w)
Supplement: Supplementary file 1 — Supplementary Information [file 41467_2022_30400_MOESM1_ESM.pdf]

Supplementary Information for

N-type fast inactivation of a eukaryotic voltage-gated sodium channel

Authors
Jiangtao Zhang, Yiqiang Shi, Junping Fan, Huiwen Chen, Zhanyi Xia, Bo Huang, Juquan Jiang, Jianke Gong, Zhuo Huang, Daohua Jiang

This file contains Supplementary Figure 1-6 and Table 1-2.

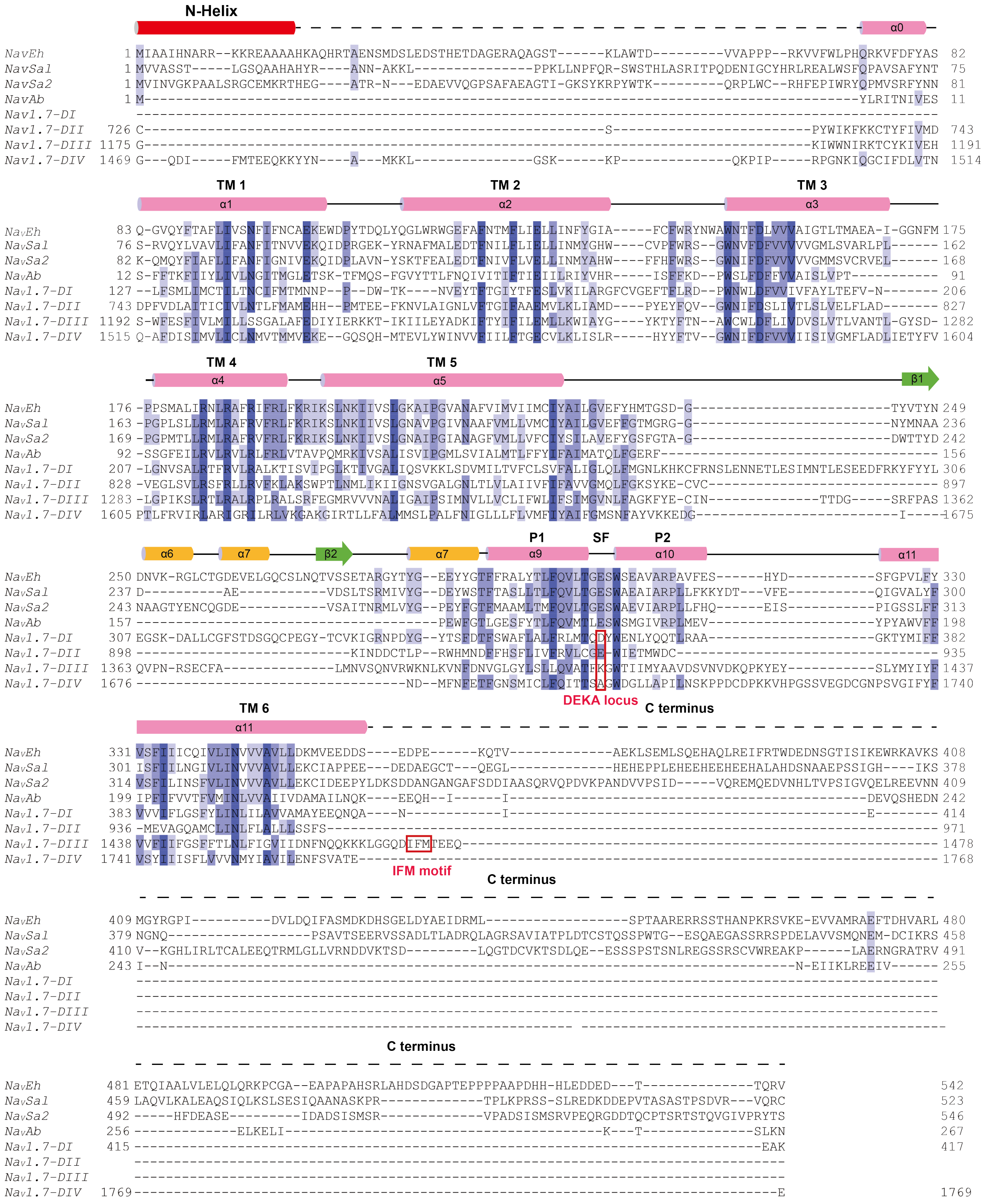

Supplementary Figure 1 Sequence alignment. Sequence alignment of Na_VEh with the other two sodium channels from coccolithophores *Scyphosphaera apsteinii* (Na_VSa1: CAMPEP_0119314838 and Na_VSa2: CAMPEP_0119345692) and human $Na_V1.7$(Uniprot: Q15858).

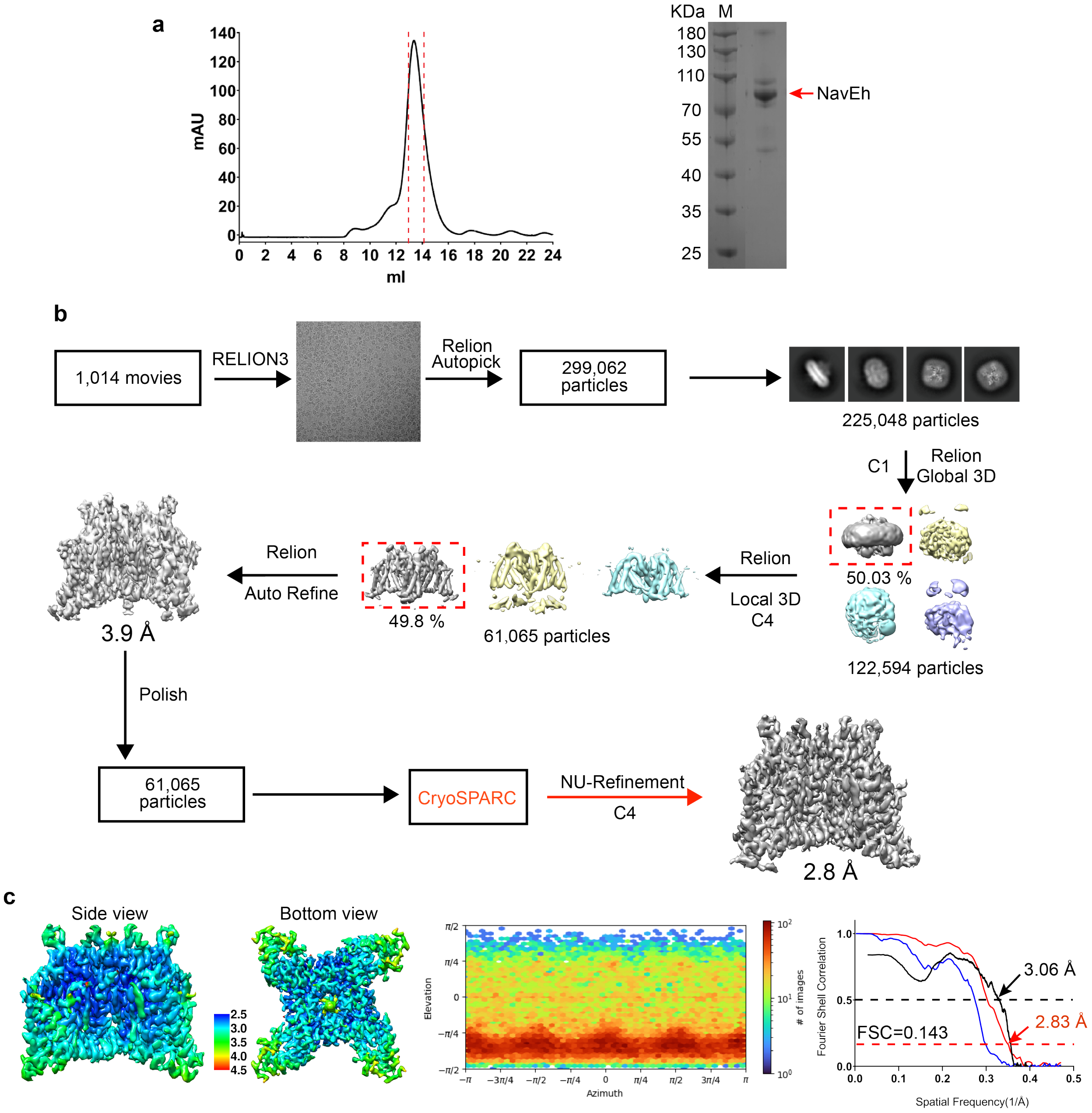

Supplementary Figure 2 Purification of the Na$_V$Eh and cryo-EM data process. **a.** A representative size exclusion chromatogram profile of purified Na$_V$Eh. Peak fractions labelled by red dashed lines were collected and concentrated for cryo-EM study. The purified Na$_V$Eh sample was stained with Coomassie brilliant blue on SDS-PAGE gel. Red arrow indicated the band for Na$_V$Eh. The experiments were repeated independently with more than 3 times with similar results. **b.** Cryo-EM data process of Na$_V$Eh. Raw movies were motion corrected, followed by 2D classification and 3D classification. The best class from last 3D classification contained a total of 61,065 particles, which were auto-refined and polished in Relion. The polished particles were imported into cryoSPARC for the final non-uniform refinement. **c.** Local resolution distribution for Na$_V$Eh map (left). Angular distribution of Na$_V$Eh map (middle). FSC of the final map, calculated between two independently refined half-maps before (blue) and after (red) post-processing. The FSC curve calculated between the cryo-EM density map and the model shown in black (right). Source data are provided as a Source Data file.

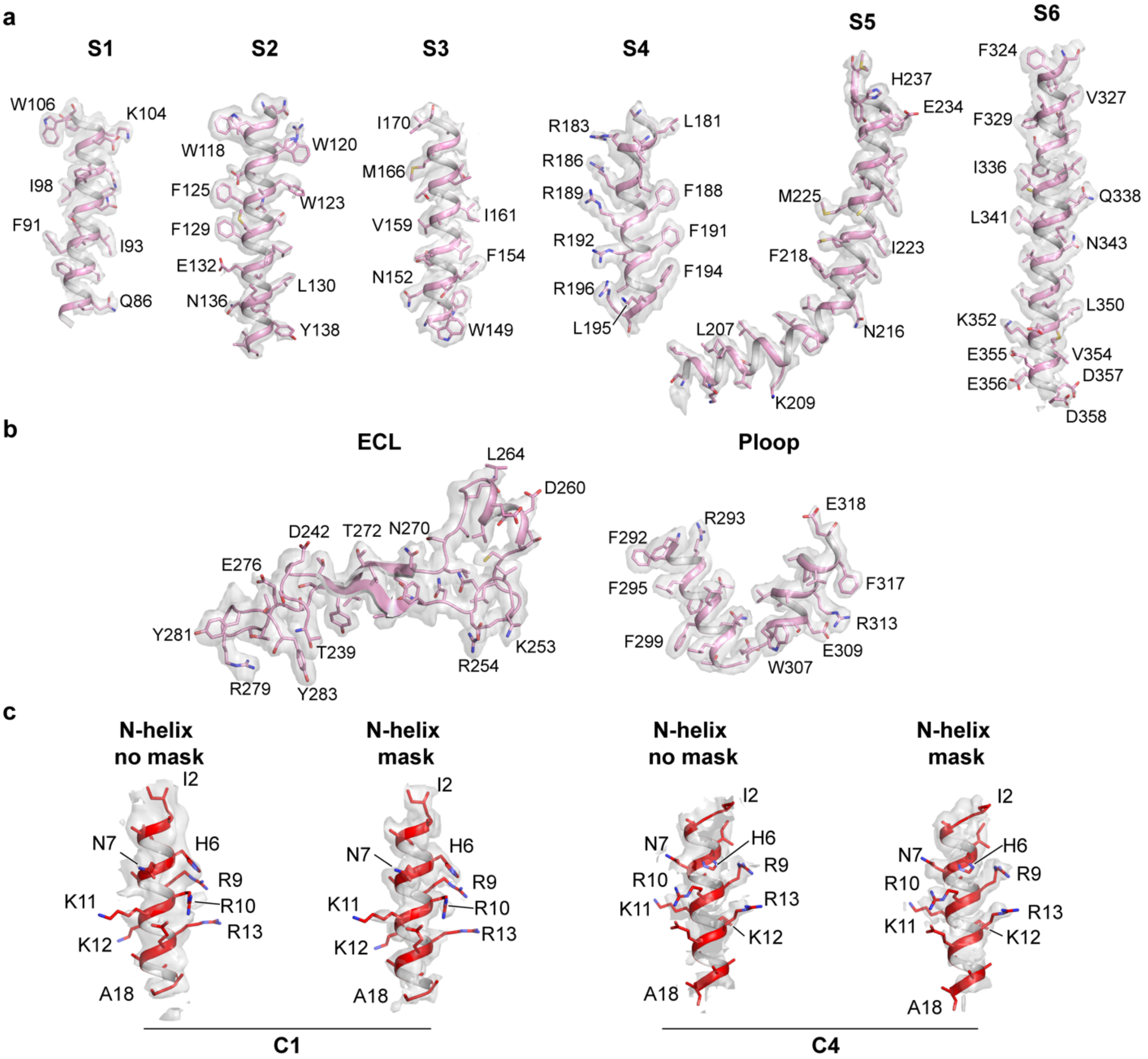

Supplementary Figure 3 EM maps for Na$_V$Eh. **a.** EM map for S1-S6 of Na$_V$Eh. **b.** EM map for ECL and p-loop of Na$_V$Eh. **c.** EM map for N-helix of Na$_V$Eh with C1- (left) and C4-symmetry (right) imposed. Structural elements are labelled and side-chains are shown as sticks.

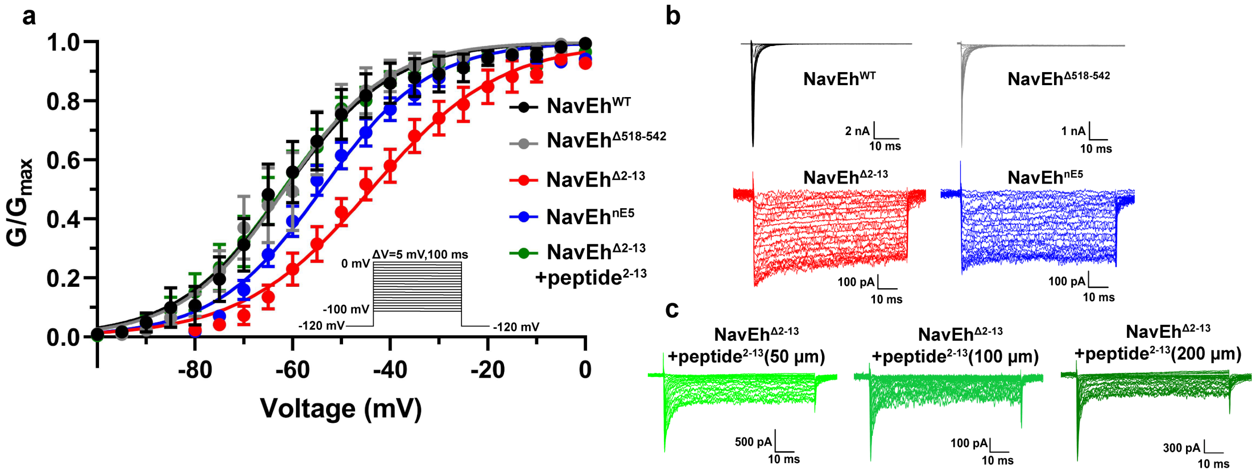

Supplementary Figure 4 Functional characteristics of Na$_V$Eh variants. **a.** Normalized conductance-voltage (G/V) relationship for Na$_V$Eh variants. For measuring G/V curve, Na$_V$Eh variants transfected HEK293T cells were measured with 100-ms depolarizing pulses between −100 mV and 20 mV in steps of 5 mV from a holding potential of −150 mV. The resulting $V_{1/2}$ for Na$_V$Eh^{WT}, Na$_V$Eh$^{Δ518\text{-}542}$, Na$_V$Eh$^{Δ2\text{-}13}$, Na$_V$Eh$^{Δ2\text{-}13}$+peptide$^{2\text{-}13}$ and Na$_V$Eh^{nE5} are –61.5 ± 2.0 mV (n=15), –61.6 ± 2.2 mV (n=12), –43.9 ± 2.2 mV (n=10), –61.9 ± 7.5 mV (n=7), and –54.0 ± 1.3 mV (n=7), respectively. Data are mean +/- SEM. **b.** Representative current traces for Na$_V$Eh variants. A family of current traces from panel **a** were presented for Na$_V$Eh^{WT}, Na$_V$Eh$^{Δ518\text{-}542}$, Na$_V$Eh$^{Δ2\text{-}13}$ and Na$_V$Eh^{nE5}, respectively. **c.** Fast inactivation of Na$_V$Eh$^{Δ2\text{-}13}$ is restored by the peptide$^{2\text{-}13}$. A family of current traces of Na$_V$Eh$^{Δ2\text{-}13}$ with intracellular application of the peptide$^{2\text{-}13}$ at 50 μM, 100 μM, and 200 μM, respectively. Source data are provided as a Source Data file.

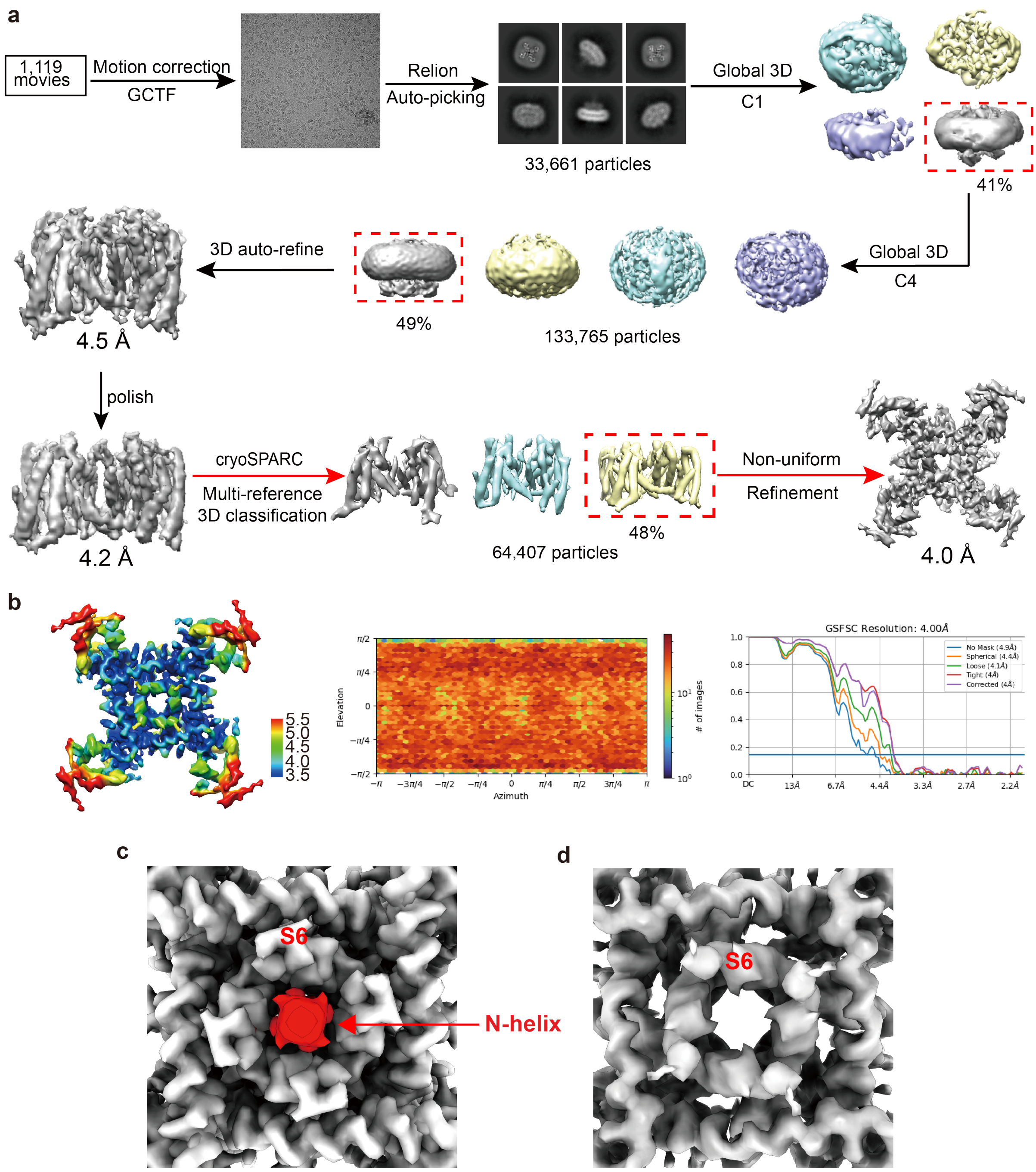

Supplementary Figure 5 Cryo-EM data processing of $Na_VEh^{\Delta 2\text{-}13}$. **a.** Data processing of $Na_VEh^{\Delta 2\text{-}13}$ following similar procedure as Na_VEh^{WT}. Particle picking, 2D and 3D classification were performed in Relion. The final class of 64,407 particles were imported into cryoSPARC and refined to 4.0 Å. **b.** Local resolution distribution (left), particle angular distribution (middle) and FSC curve (right) of $Na_VEh^{\Delta 2\text{-}13}$ map. **c-d.** Activation gate of Na_VEh^{WT} map and $Na_VEh^{\Delta 2\text{-}13}$ map, respectively. The EM density for N-helix is highlighted in red.

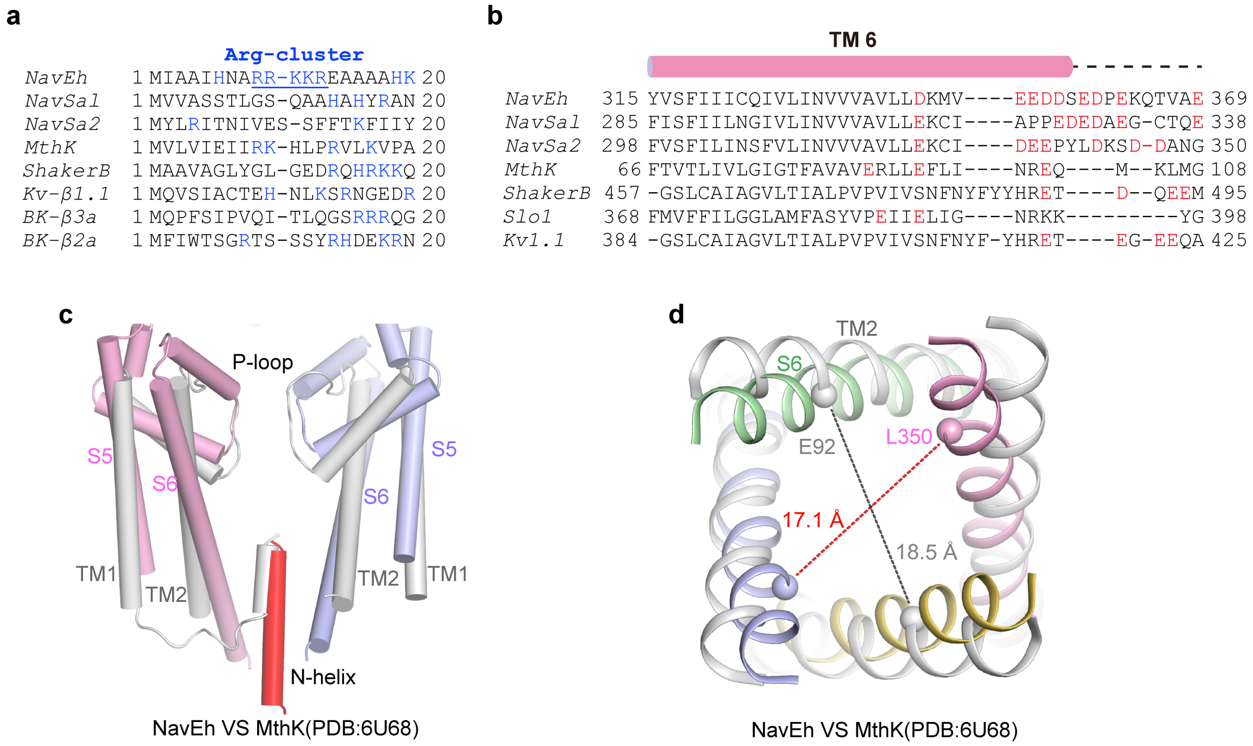

Supplementary Figure 6 Gate comparison of Na$_V$Eh and MthK. **a.** Sequence alignment of the N-terminus responsible for N-type inactivation from Na$_V$Eh (Accession number CAMPEP_01 87645740), Na$_V$Sa1 (Accession number CAMPEP_01 19314838), Na$_V$Sa2 (Accession number CAMPEP_01 19345692), MthK (accession number CEP 36137), Shaker B (accession number CAA 29917), Kv-β1.1 (accession number CAA 50000), BK-β3a (accession number NP_741979) and BK-β2a (accession number NP_001265840). **b.** Sequence alignment of the pore-lining helix from Na$_V$Eh, Na$_V$Sa1, Na$_V$Sa2, MthK, Shaker B, Slo1 (accession number NM_001014797), K$_V$1.1 (accession number NP_000208). Basic and acidic amino acid are colored by blue and red, respectively. **c.** Pore domain structural comparison between Na$_V$Eh (pink and light blue) and Mthk (PDB ID 6U68, gray), S5 and S6 helices were used for superimposition. **d.** Gate comparison between Na$_V$Eh and Mthk. Distances were measured from alpha carbon atoms of opposing residues.

Supplementary Table 1. Na$_V$Eh protein and gene sequence.

Species	Strain	Name	Protein ID
Emiliania huxleyi	**CCMP379**	**Na$_V$Eh (EhEUKCATB1)**	**CAMPEP_0187654740**

Protein Sequence

MIAAIHNARRKKREAAAAHKAQHRTAENSMDSLEDSTHETDAGERAQAGSTKLAWTDVVAPPPRKVVFWLPHQR
KVFDFYASQGVQYFTAFLIVSNFIFNCAEKEWDPYTDQLYQGLWRWGEFAFNTMFLIELLINFYGIAFCFWRYNWA
WNTFDLVVVAIGTLTMAEAIGGNFMPPSMALIRNLRAFRIFRLFKRIKSLNKIIVSLGKAIPGVANAFVIMVIIMCIYAILG
VEFYHMTGSDGTYVTYNDNVKRGLCTGDEVELGQCSLNQTVSSETARGYTYGEEYYGTFFRALYTLFQVLTGES
WSEAVARPAVFESHYDSFGPVLFYVSFIIICQIVLINVVVAVLLDKMVEEDDSEDPEKQTVAEKLSEMLSQEHAQLREI
FRTWDEDNSGTISIKEWRKAVKSMGYRGPIDVLDQIFASMDKDHSGELDYAEIDRMLSPTAARERRSSTHANPKRS
VKEEVVAMRAEFTDHVARLETQIAALVLELQLQRKPCGAEAPAPAHSRLAHDSDGAPTEPPPPAAPDHHHLEDDED
TTQRV

Codon-optimized gene of NavEh

ATGATCGCCGCCATCCACAACGCTAGAAGAAAGAAGCGGGAGGCCGCTGCCGCCCACAAGGCTCAGCACAG
AACCGCCGAGAACAGCATGGATAGCCTGGAGGACTCCACCCACGAGACCGACGCCGGCGAGAGAGCCCAA
GCCGGCAGCACCAAACTGGCCTGGACCGACGTGGTGGCCCCCCCTCCTAGAAAGGTGGTGTTCTGGCTGCC
CCATCAGAGAAAGGTGTTCGACTTCTACGCTAGCCAAGGCGTGCAGTACTTCACCGCCTTCCTGATCGTGAGC
AACTTCATCTTCAACTGCGCCGAGAAGGAGTGGGACCCCTACACCGATCAGCTGTACCAAGGCCTGTGGAGA
TGGGGCGAGTTCGCCTTCAACACCATGTTCCTGATCGAGCTGCTGATCAACTTCTACGGCATCGCCTTCTGCT
TCTGGAGATACAACTGGGCCTGGAACACCTTCGACCTGGTGGTCGTGGCCATCGGCACCCTGACCATGGCC
GAGGCCATCGGCGGCAACTTCATGCCCCCTAGCATGGCCCTGATCAGAAACCTGAGAGCCTTCAGAATCTTC
AGACTGTTCAAGAGAATCAAGAGCCTGAACAAGATCATCGTGAGCCTGGGCAAGGCCATCCCCGGCGTGGCC
AACGCCTTCGTGATCATGGTGATCATCATGTGCATCTACGCCATCCTGGGCGTGGAGTTCTACCACATGACCG
GCAGCGACGGCACCTACGTGACCTACAACGACAACGTGAAGAGAGGCCTGTGCACCGGCGACGAGGTGGA
GCTGGGGCAGTGCAGCCTGAATCAGACCGTGAGCAGCGAGACCGCTAGAGGCTACACCTACGGCGAGGAGT
ACTACGGCACCTTCTTCAGAGCCCTGTACACCCTGTTCCAAGTGCTGACCGGCGAGAGCTGGAGCGAGGCC
GTGGCTAGACCCGCCGTGTTCGAGAGCCACTACGACAGCTTCGGCCCCGTGCTGTTCTACGTGAGCTTCATC
ATTATCTGTCAGATCGTGCTGATCAACGTGGTCGTGGCCGTCCTGCTGGACAAGATGGTGGAGGAGGACGAT
AGCGAGGACCCCGAGAAGCAGACCGTGGCCGAGAAGCTGAGCGAGATGCTGAGCCAAGAGCACGCTCAGC
TGAGAGAGATCTTCAGAACCTGGGACGAGGACAACAGCGGCACCATCAGCATCAAGGAGTGGAGAAAGGCC
GTGAAGAGCATGGGCTACAGAGGCCCCATCGACGTGCTGGATCAGATCTTCGCTAGCATGGACAAGGACCAC
AGCGGCGAGCTGGACTACGCCGAGATCGACAGAATGCTGAGCCCCACCGCCGCCCGGGAGAGAAGAAGCA
GCACCCACGCCAACCCCAAGAGAAGCGTGAAGGAAGAGGTGGTCGCCATGAGAGCCGAGTTCACCGACCAC
GTGGCTAGACTGGAGACACAGATCGCCGCCCTGGTGCTCGAGCTGCAGCTCCAAAGAAAACCCTGCGGGGC
CGAAGCTCCCGCTCCCGCCCACTCCCGGCTGGCCCACGATAGCGATGGCGCCCCTACAGAGCCCCCTCCTC
CCGCTGCTCCCGATCACCATCACCTGGAGGACGACGAGGACACCACACAGAGAGTG

Supplementary Table 2. Cryo-EM data collection, refinement and validation statistics

	Na_VEh (EMD-33016) (PDB: 7X5V)	$Na_VEh^{\Delta 2\text{-}13}$ (EMD-33017)
**Data collection and processing**		
Magnification	105,000 ×	105,000 ×
Voltage (kV)	300	300
Electron exposure ($e^-/Å^2$)	60	60
Defocus range (μm)	−1.2 ~ −2.2	−1.2 ~ −2.2
Pixel size (Å)	1.04	1.04
Symmetry imposed	C4	C4
Initial particle images (no.)	299,062	579,023
Final particle images (no.)	61,065	64,407
Map resolution (Å)	2.83	4.02
FSC threshold	0.143	0.143
Map resolution range (Å)	2.5 ~ 4.5	3.5 ~ 5.5
**Refinement**		
Initial model used (PDB code)	Alphafold2 model	
Model resolution (Å)	3.06	
FSC threshold	0.5	
Map sharpening *B* factor ($Å^2$)	-84.8	
Model composition		
Non-hydrogen atoms	9,348	
Protein residues	1,160	
Ligands	0	
B factors ($Å^2$)		
Protein	51.43	
R.m.s. deviations		
Bond lengths (Å)	0.006	
Bond angles (°)	0.757	
Validation		
MolProbity score	2.83	
Clashscore	18.8	
Poor rotamers (%)	1.60	
Ramachandran plot		
Favored (%)	93.75	
Allowed (%)	6.25	
Disallowed (%)	0.00	
